# Whole blood RNA profiling in cats dissects the host immunological response during recovery from feline infectious peritonitis

**DOI:** 10.1371/journal.pone.0332248

**Published:** 2025-09-12

**Authors:** Katharina Zwicklbauer, Pilar Grassl, Martin Alberer, Laura Kolberg, Nina A. Schweintzger, Sonja Härtle, Kaspar Matiasek, Regina Hofmann-Lehmann, Katrin Hartmann, Caroline C. Friedel, Ulrich von Both

**Affiliations:** 1 LMU Small Animal Clinic, Centre for Clinical Veterinary Medicine, LMU Munich, Munich, Germany; 2 Institute for Informatics, LMU Munich, Munich, Germany; 3 Division of Paediatric Infectious Diseases, Dr. von Hauner Children’s Hospital, University Hospital, LMU Munich, Munich, Germany; 4 Department of Pediatrics and Adolescent Medicine, Division of General Pediatrics, Medical University of Graz, Graz, Austria; 5 Center for Medical Research, Medical University of Graz, Graz, Austria; 6 Department of Veterinary Sciences, AG Immunology, LMU Munich, Planegg, Germany; 7 Section of Clinical and Comparative Neuropathology, Institute of Veterinary Pathology, Centre for Clinical Veterinary Medicine, LMU Munich, Munich, Germany; 8 Department of Clinical Diagnostics and Services, Clinical Laboratory, and Center for Clinical Studies, Vetsuisse Faculty, University of Zurich, Zurich, Switzerland; 9 German Center for Infection Research (DZIF), Partner Site Munich, Munich, Germany; Universidad Cooperativa de Colombia, COLOMBIA

## Abstract

Feline infectious peritonitis (FIP) is caused by infection with the feline coronavirus (FCoV) and is fatal if left untreated. In most cats, FCoV primarily infects the gastrointestinal tract and remains asymptomatic or causes only mild enteritis, with only a small proportion of infected cats developing FIP. An excessive and harmful immune response leading to characteristic (pyo)granulomatous phlebitis is believed to play a key role in the development of FIP, along with complex interactions between host and viral factors. Our research group recently demonstrated successful treatment of cats with naturally occurring FIP using the antiviral nucleoside analogue GS-441524. Treatment led to complete recovery without any relapses for a follow-up period of one year, demonstrating both a short- and long-term cure. To investigate differential gene expression and corresponding molecular pathways in cats with FIP before, during, and after antiviral treatment, RNA sequencing was performed on full blood samples of 18 cats treated successfully in a prospective study. Samples were analyzed before treatment, at different timepoints while on treatment with GS-441524 and after completion of treatment. Additionally, gene expression profiles were compared to 12 healthy FCoV-infected control cats and 5 healthy uninfected control cats. The results revealed both a widespread dysregulation of the blood RNA signature in cats with FIP as well as its rapid normalization within the first week of treatment. Significant changes were already apparent within the first two days of treatment. The results of the present study suggest that elimination of the virus from the blood leads to rapid control and subsequent normalization of the damaging immune response, a finding that corresponds well to the clinical response to treatment. This study illustrates the host response to treatment at the molecular level and provides further evidence that a shorter treatment duration than the 84 days predominantly practiced is sufficient.

## Introduction

Coronaviruses are a large family of RNA viruses infecting a variety of mammalian and avian hosts, causing severe disease in some species [1]. Their high genetic diversity promotes the frequent emergence of new variants with changes in cell tropism or host range, enabling interspecies or zoonotic transmission. Notable examples include Severe Acute Respiratory Syndrome Coronavirus (SARS-CoV), Middle East Respiratory Syndrome Coronavirus (MERS-CoV), and Severe Acute Respiratory Syndrome Coronavirus 2 (SARS-CoV-2), the virus responsible for Coronavirus Infectious Disease 2019 (COVID-19) [2–5]. Among animal species, the feline coronavirus (FCoV) is particularly important. It is primarily transmitted between cats via the fecal-oral route, where it enters enterocytes and typically causes no clinical signs or only mild intestinal diseases [6–9]. However, in a subset of infected cats (approximately 5–10%), an infection with FCoV can lead to immune-mediated disease known as feline infectious peritonitis (FIP), which is always fatal if left untreated, with a median survival time of only 8–9 days after diagnosis [10,11]. This progression is most likely caused by mutations in the spike gene (S gene) of FCoV, which enables the virus to infect monocytes/macrophages, to replicate successfully within them, and to spread systemically [12]. The exact reasons for the development of FIP remain unclear, but it is believed to be the result of a combination of viral and host factors [13,14]. The clinical presentation of FIP is characterized by (pyo)granulomatous phlebitis, often accompanied by extensive pyogranulomatous lesions and, in many cases, by cavitary effusions [15,16].

The application of next-generation sequencing techniques for RNA sequencing (RNA-seq) has greatly advanced the understanding and characterization of cellular expression profiles during pathogen infection. This approach has demonstrated its utility in shedding light on the pathogenesis of numerous viruses [17,18], including the feline immunodeficiency virus (FIV) [19,20]. The growing access to complete genome sequences for various model organisms enhances the value of host transcriptome analysis to uncover mechanisms underlying virus pathogenesis and host response to viral infections.

So far, RNA-seq in the context of FIP has only been applied *in vitro* to peritoneal macrophages [21–23] as well as post-mortem mesenteric lymph nodes of cats with FIP to analyze immune responses at the organ level [24]. The latter study showed that cats with FIP exhibited stronger expression of humoral immunity and higher activation of pro-inflammatory pathways compared to cats with an infection of the non-mutated wildtype of FCoV, which showed lower enrichment of humoral immunity pathways. Recently, a proteomic approach was used to compare plasma protein profiles between three distinct groups of field cats with FIP, healthy FCoV-infected and healthy, non-FCoV-infected cats [25]. This study identified enrichment of proteins associated with immune system processes in plasma profiles of cats with FIP, including innate immune response, cytokine signaling, antigen presentation, apoptosis, and vascular integrity. However, until recently, only the progression of FIP (and not the cure) could be studied because the disease was invariably fatal [10,11]. Moreover, none of the previously mentioned studies used full blood samples for RNA-seq analysis.

Recently, human antiviral medications have become available that can be used to successfully treat FIP. Most of these had not been trialed or licensed. Among these antivirals, the nucleoside analogue GS-441524, the active compound of Remdesivir [26], has shown remarkable success in several prospective studies [27–41]. We recently conducted the first prospective clinical trial including 18 cats with naturally occurring FIP, administering the oral antiviral drug Xraphconn^®^ (Mutian Life Sciences Limited, China) containing the active ingredient GS-441524 [31]. Cats received treatment once daily for 84 days and were followed for up to one year after treatment initiation (day 336; 9 months after completion of the antiviral treatment) [38]. All 18 cats were successfully cured after 84 days of treatment (day 83) and remained healthy within the entire follow-up period.

The aim of the present study was to perform RNA-seq on feline full blood samples taken before, during and after completion of treatment to analyze the molecular RNA signatures of cats with FIP. This allowed for tracking of changes in gene expression from the time of diagnosis through treatment to complete recovery and highlighted key immune pathways involved in disease progression, treatment response, and recovery. These novel findings shed light on the immune mechanisms underlying FIP and pave the way for a better understanding and treatment of this previously deadly condition.

## Results

### FIP leads to genome-wide expression changes that are rapidly normalized by treatment

**Fig 1:**
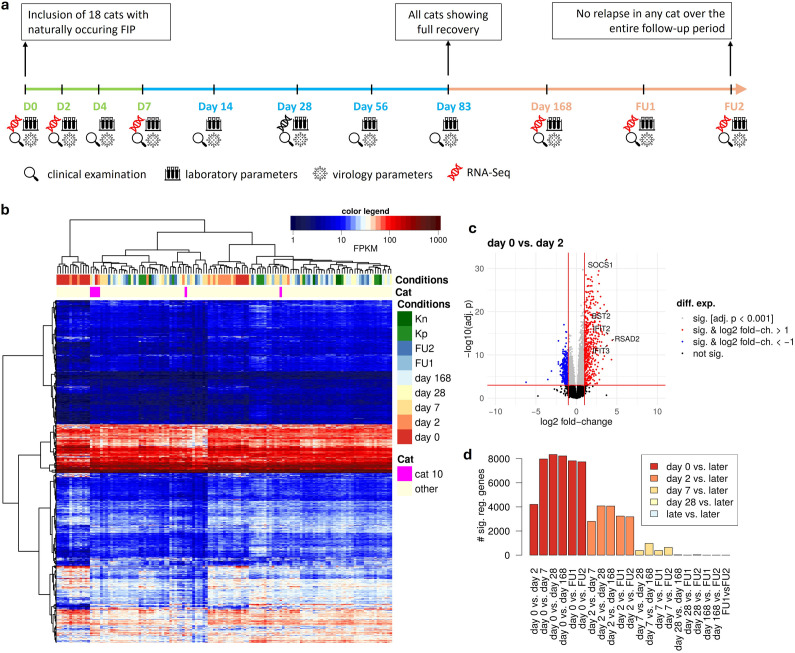
Overview of the study design and resulting RNA changes. (a) Timeline visualizing the study course. From days 0–7 the cats were hospitalized (green; D0 = before treatment, D1–7 = hospitalization during treatment) and were treated at home for the remaining days until day 83 (blue = during treatment), with re-checks on days 14, 28, and 56. After the end of treatment (orange), the cats were followed at 3-month intervals for a total of one year from the start of the treatment (days 168, 252 [follow-up 1(FU1)], and 336 [follow-up 2 (FU2)]). Timepoints for clinical examinations, evaluation of laboratory parameters, measurement of virology parameters (including viral loads in blood, effusion and feces, anti-FCoV antibodies) and sampling for RNA-seq are indicated. (b) Heatmap showing log10 FPKM values for 9,142 genes with expression (FPKM >0.5) in all samples. Genes (rows) and samples (columns) were clustered hierarchically using Euclidean distances and Ward’s clustering criterion [43]. The color bar on top of the heatmap indicates the mapping of colors to FPKM values. Timepoints of treatment or control cats are indicated on top of the heatmap (=conditions) and samples of cat 10 are particularly highlighted (= cats). (c) Volcano plot for the differential gene expression analysis between day 0 and day 2. Genes strongly up- (log2 fold-change >1) or down-regulated (log2 fold-change < −1) are marked in red and blue, respectively. The 5 most strongly regulated genes with an annotated function are highlighted. (d) Number of genes significantly (adj. p < 0.001) differentially expressed between each timepoint and all later timepoints.

RNA-seq analysis was performed on whole blood samples for the 18 cats treated with GS-441524 taken at the following timepoints: before treatment (day 0), during treatment (days 2, 7, and 28 after the start of treatment), and after treatment (days 168, 252 [follow-up 1, abbreviated as FU1], and 336 [follow-up 2, abbreviated as FU2]) (see Fig 1a and S1 Table for an overview). In addition, samples of a FCoV-uninfected, negative control group were obtained at a single timepoint from 5 healthy, single-kept indoor-only cats (abbreviated as Kn), which were presented for regular health care appointments at our clinic. These cats were tested negative for anti-FCoV antibodies in serum and had no fecal shedding of FCoV, as determined by previously described methods [31,38,42]. A FCoV-infected, positive control group (abbreviated as Kp) consisted of 12 cats that were healthy cohabitating partner cats (without FIP) of the treatment study cats with FIP (see S1 Table and S2 Table for the overview of patients). At the time of sampling, viral RNA was detected in the feces of 8 of 12 (67%) healthy Kp cats. The FCoV RNA loads varied, ranging from approximately 160,000 to nearly 75 million copies per gram of feces. Neither the proportion of cats shedding viral RNA in feces nor the respective viral loads differed significantly between cats with FIP at the beginning of therapy (61%; days 0–2) and the companion Kp cats. All 12 cats had anti-FCoV antibodies in serum [42].

Gene expression analysis (quantified as fragments per kilobase of transcript per million fragments mapped [FPKM]) for cats with FIP across all timepoints and Kn and Kp cats, showed that day 0 samples exhibited largely distinct expression profiles, mostly clustering separately from later timepoints and from healthy cats (Fig 1b). Similarly, the very early timepoints of treatment (days 2 and 7) largely clustered together and together with a group of day 0 samples that clustered separately from the other day 0 samples. Strikingly, day 0 and most (4 of 5) treatment samples of cat 10 clustered separately from other day 0 and early treatment samples but rather with Kn, Kp, and late treatment samples. This is consistent with the clinical representation of cat 10 on day 0. This cat had no clinical or laboratory changes, which can be typically altered in cats with FIP [44,45], and exhibited only a minimal amount of free fluid in the abdomen during the ultrasonographic examination on day 0. The cat was clinically healthy, and FIP was diagnosed by chance by detecting FCoV antigen by immunohistochemistry (IHC) in an altered lymph node found and removed during neutering.

To account for the differences between cats with FIP already at day 0, we used a multi-factor design for differential gene expression analysis that takes into account which samples corresponded to the same cat. This recovers consistent changes in RNA levels for each gene during treatment, even if overall RNA levels differ between cats. Differential gene expression analysis was performed to identify differentially expressed genes (DEGs, multiple testing adjusted p-value [adj. p]<0.001) between all timepoints of treatment as well as between day 0 cats compared to Kn and Kp cats. The results of this analysis showed that 9,763 (~75%) of 12,935 analyzed genes with mean read count >10 were significantly differentially expressed between day 0 and any of the later timepoints, indicating an almost genome-wide shift in gene expression. It should be noted, however, that bulk RNA-seq performed here cannot distinguish between changes in gene expression for individual cells and changes in cell type composition of the respective blood samples. We therefore use the term blood RNA signature in the following to denote the combined effect of gene expression changes and alterations in cell type composition.

**Fig 2:**
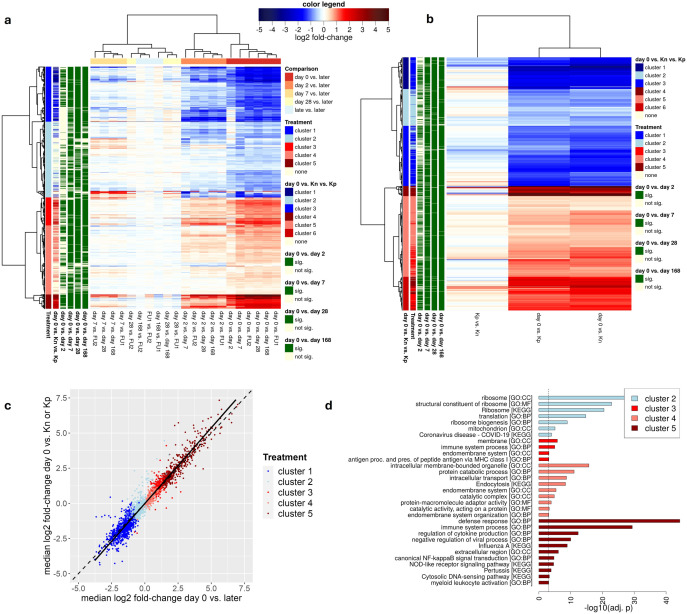
Differential gene expression analysis. (a) Heatmap showing log2 fold-changes for DEGs identified between day 0 and days 2, 7, 28, or 168. The color bar on top of the heatmap indicates the mapping of colors to log2 fold-changes. Genes (rows) and pairwise comparison (columns) were clustered hierarchically using Euclidean distances and Ward’s clustering criterion. Identified gene clusters are indicated to the right of the gene dendrogram (= Treatment). The clusters identified for the DEGs in the comparison between cats with FIP at day 0, Kn and Kp cats (see b) are also indicated (= day 0 vs. Kn vs. Kp), and whether a gene is statistically significant in the comparison between day 0 and day 2, day 7, day 28 or day 168, respectively. (b) Heatmap as in (a) showing the log2 fold-changes for DEGs identified between day 0, Kn and Kp cats and results of the hierarchical clustering analysis on these genes. (c) Scatterplot comparing median log2 fold-changes between day 0 and Kn or Kp cats (y-axis) against median log2 fold-changes between day 0 and any of the later timepoints (x-axis). Points are colored according to the clusters identified in (a). The dashed line indicates results of a diagonal while the solid line indicates results of a linear regression analysis. (d) Overview of significantly enriched GO terms and KEGG pathways for the five clusters identified in (a). The dashed vertical line indicates the p-value cutoff. For the GO, only driver terms selected by g:Profiler are shown, whereas for KEGG all significant pathways are shown. For each cluster, enriched functions are sorted by adj. p.

Most changes occurred rapidly early in treatment with ~4,000 DEGs observed on day 2 compared to day 0 (Fig 1c) and >8,000 genes differentially expressed within the first month of treatment (Fig 1d). In contrast, far fewer changes were observed after the first week (1,170 DEGs between day 7 and later timepoints) and even fewer after the first month (36 DEGs between day 28 and later timepoints), consistent with the clinical presentation of the cats showing rapid recovery [31]. We then focused on the 9,605 DEGs identified between day 0 and days 2, 7, 28, or 168 and performed hierarchical clustering analysis of log2 fold-changes between any 2 timepoints to identify groups of genes with common RNA signature changes (Fig 2a). This identified 5 distinct gene clusters, representing either genes that were down-regulated (clusters 1 & 2) or up-regulated (clusters 3–5) in day 0 cats compared to later timepoints (Fig 2a and S3 Table). Cluster 1 (2,185 genes) consisted of genes that were strongly down-regulated at day 0 and to a large degree significantly less down-regulated already at day 2. In contrast, cluster 2 genes (3,002 genes) were only moderately down-regulated at day 0 with significant changes commonly observed not before day 7. On the other hand, cluster 5 (571 genes) contained genes highly up-regulated in day 0 cats that responded quickly to treatment, with 498 (87%) of these genes already significantly less up-regulated at day 2. Cluster 3 (1,106 genes) included genes moderately up-regulated at day 0 and cluster 4 (2,741 genes) weakly up-regulated genes that responded more slowly, but whose up-regulation was generally also reduced by day 7.

Differential gene expression analysis between cats with FIP (day 0), Kn and Kp identified 5,714 DEGs, of which 5,675 (99%) also significantly changed expression during treatment. Here, cat 10 was excluded as it clustered with healthy cats in Fig 1b. No genes were differentially expressed between Kn and Kp cats. Hierarchical clustering analysis identified 6 groups of DEGs (Fig 2b and S4 Table): (i) clusters 1–3, which were down-regulated to varying degrees in sick cats compared to healthy cats, with clusters 1 and 3 largely matching cluster 1 from the treatment analysis and cluster 2 largely matching cluster 2 from treatment; (ii) clusters 4–6, which were up-regulated in sick cats compared to healthy cats to varying degrees. Here, cluster 4 was most strongly up-regulated in sick cats and largely matched cluster 5 from treatment, while cluster 6 was moderately up-regulated and matched clusters 5 and 3 from treatment and cluster 5 was least up-regulated and matched clusters 3 and 4 from treatment (Fig 2b). Generally, DEGs identified during treatment showed the same trend in the comparison of sick vs. healthy cats, even if changes were not statistically significant (Fig 2c). Furthermore, most genes strongly differentially expressed in treatment (cluster 5) were also significant when comparing sick and healthy cats (Fig 2a). The relatively low number of samples for control cats likely explains why less strongly pronounced changes did not reach the level of statistical significance for sick vs. healthy cats. In summary, the massive changes in RNA blood signatures in the first month of treatment compared to day 0 resulted from a rapid normalization of RNA blood signatures and reflect differences observed between sick and healthy cats.

### FIP is associated with a strong immune response in whole blood

**Fig 3:**
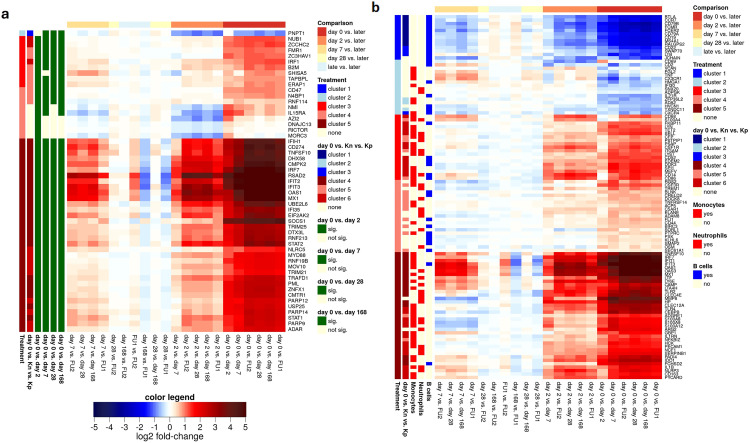
Differential gene expression for ISGs and cell type markers. Heatmaps showing log2 fold-changes for (a) vertebrate core ISGs differentially expressed during treatment and (b) cell type marker genes for monocytes, neutrophils and B cells. Order of genes (rows) and samples (columns) were taken from the hierarchical clustering in Fig 2a. For the description of the heatmaps, see caption to Fig 2a. In (b) the cell type for which a gene is a marker gene is also indicated.

To identify characteristics of DEGs, functional enrichment analysis was performed for the 5 clusters of DEGs identified during treatment with g:Profiler [46] for Gene Ontology (GO) terms and KEGG pathways (S5 Table). This analysis showed a strong enrichment (adj. p < 0.001) of genes involved in the (innate) immune defense response among DEGs strongly upregulated at day 0 (cluster 5, Fig 2d). This included negative regulation of viral processes, in particular the pattern recognition receptor signaling pathway, regulation of cytokine production and the response to type I interferon (IFN) (S5 Table). Accordingly, strongly up-regulated genes (cluster 5) contained several interferon-stimulated genes (ISGs), including 35 of 62 core ISGs found to be up-regulated in response to IFN across vertebrates (including human, rat, cow, sheep, pig, horse, dog, little brown bat, large flying fox, fruit bat, and chicken) by Shaw and colleagues [47] (~27-fold enriched, Fisher’s exact test p-value 3.92 x 10^−29^, Fig 3a). Well-known antiviral ISGs strongly up-regulated in sick cats included MX1, OAS1, −2, −3, -L, ISG20, IFIT1, −2, −3, −5 and RIG-I. Consistent with cluster 5 representing a general antiviral response, the KEGG Influenza A pathway was also highly enriched in cluster 5 as well as genes up-regulated in human cells in response to infection with several respiratory viruses [48], including SARS-CoV-2, RSV, and human parainfluenza virus 3.

Genes moderately up-regulated at day 0 (cluster 3) were also enriched for immune responses but less pronounced, while weakly up-regulated genes (cluster 4) were enriched for protein catabolic process, which includes a number of ubiquitin-specific proteases but also other enzymes involved in ubiquitin-dependent protein catabolic processes. In contrast, no enrichment for GO terms or KEGG pathways was found for genes strongly down-regulated in day 0 (cluster 1), but weakly down-regulated genes (cluster 2) were enriched for ribosome subunits. In summary, these results indicate a massive up-regulation of the antiviral immune response in cats with FIP, which is dramatically reversed already by 2 days of effective treatment.

**Fig 4:**
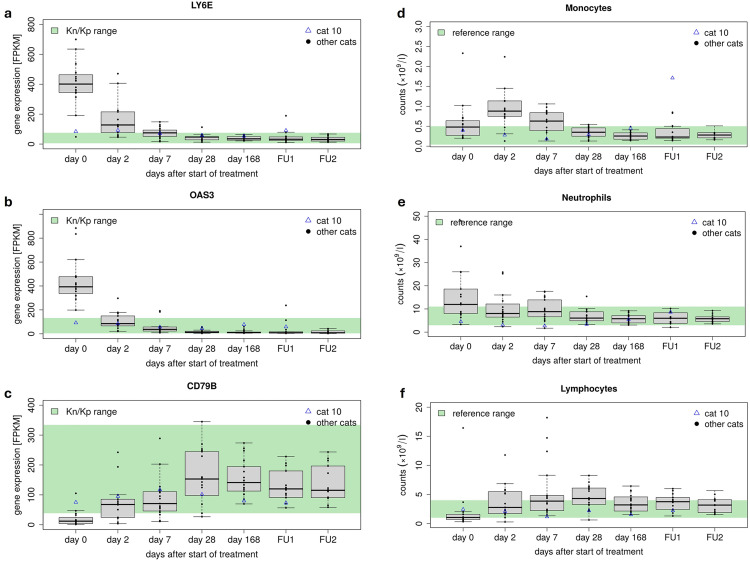
Selected cell type marker expression and laboratory parameters before, during and after antiviral treatment. (a-c) Box plots showing distribution of gene expression for monocyte and neutrophil markers LY6E and OAS3 and B cell marker CD79B across cats with FIP for each timepoint of treatment. Boxes indicate the range between the second and the third quantile, whiskers include all values within the 1.5 interquartile range, dots represent the values (with outliers outside a box). The horizontal line in the boxes represents the median. Values for cat 10 are not included in the box plots but are shown separately as blue rectangles. The range observed in healthy Kn or Kp cats is indicated by the green backgrounds. (d-f) Box plots showing absolute neutrophil, monocyte and lymphocyte counts for cats with FIP as in (a-c).

To investigate whether these RNA signature changes in cats with FIP represent a switch in cell type composition of the blood, enrichment for human cell type marker genes from PanglaoDB, a database of processed single cell RNA sequencing datasets for the scientific community, was also analyzed [49]. This revealed an enrichment for monocyte, neutrophil and Kupffer cell markers among genes strongly up-regulated in sick cats (cluster 5) and for B cells (both B cells in general and naïve, memory and plasma B cells) in strongly down-regulated genes (cluster 1) (Fig 3b). In particular, the enrichment for monocyte and neutrophil markers as well as B cells was also observed for cluster 5 and cluster 1 genes, respectively, that were already significantly differentially expressed at day 2. Marker genes showing high expression at day 0 and much lower levels in treated/control cats include IFIT1, OAS1, LY6E, MX1 for monocytes and OAS3 for neutrophils (Figs 3b, 4a,b). These genes also showed increased RNA levels at day 0 for cat 10, though not as elevated as in day 0 samples of other cats and generally not elevated beyond levels observed in control cats. B cell-specific markers with a strong reduction at day 0 and higher levels in treated/control cats include CD79A and CD79B, which form the B-cell antigen receptor (Figs 3b, 4c). Comparison to routine hematology examinations for the 18 cats confirmed a switch in cell type composition under treatment: Increased monocyte counts (monocytosis) in blood were present in 15/18 cats before the beginning of treatment (i.e., at days 0 and 2), in 10 cats on day 7, but only in 2 cats on day 28 and the follow-up period (Fig 4d). Increased neutrophil counts (neutrophilia) were observed in 10/18 cats before the beginning of treatment, in five cats on day 7, but only in one cat on day 28 and in none of the cats during the follow-up period (Fig 4e). In contrast, most cats exhibited a marked lymphopenia, i.e., low lymphocyte counts on day 0 before treatment (Fig 4f). Lymphocyte counts increased after treatment initiation, with a moderate to severe lymphocytosis evolving in 14/18 cats during the early treatment course (up until days 7 and 28). During the follow-up period, only a very mild lymphocytosis was still present in some cats. It should be noted that PanglaoDB does not include marker genes for lymphocytes in general, but only for subtypes such as B cells and T cells. T cell marker genes were not enriched in any clusters. The hematology results do not indicate such a dramatic switch in cell type composition that this alone would explain the massive changes observed in the RNA signature. For instance, while monocyte counts increased at most by <4-fold, LYE6E expression increased >13-fold. We thus conclude that the switch in blood RNA signature during FIP treatment is shaped by both a down-regulation of the immune response in individual cells and decreased and increased monocyte and B cells count, respectively.

## Discussion

In this study, we present the first RNA-seq analysis of gene expression changes during successful treatment of FIP. Here, whole blood gene expression analysis of cats with FIP over all timepoints, of clinically healthy, FCoV-uninfected, negative control cats (Kn) and of clinically healthy, FCoV-infected, positive control cats (Kp) showed a distinct RNA expression pattern of cats with FIP on day 0 (before treatment) compared to other timepoints and compared to healthy Kn and Kp cats. This revealed two key observations: first, both FIP as well as antiviral treatment of FIP led to large and significant changes in blood RNA expression profiles, with ~75% of genes exhibiting differences in RNA levels before and during treatment. Second, the RNA signature rapidly normalized within the first week of treatment, with only minimal changes observed after the first month of treatment.

Genes strongly up-regulated in the blood of the cats with FIP were predominantly involved in antiviral immune system processes. Consistent findings were reported in a recent study that employed aptamer-based proteomic assays to analyze plasma protein profiles in 19 cats naturally infected with FIP, compared to clinically healthy, FCoV-negative (n = 17) and FCoV-positive (n = 9) cats [25]. A total of 442 proteins (219 up-regulated, 223 down-regulated) were found to be significantly differentially expressed between cats with FIP and clinically healthy controls, with up-regulated proteins also involved in immune processes. Our study also showed that there were no significant changes in RNA expression between healthy Kp and Kn cats, comparable to the absence of changes in plasma protein profiles between clinically healthy FCoV negative and positive cats in the above-mentioned study [25]. Cats with a non-pathogenic enteric FCoV infection remain asymptomatic or exhibit only very mild, transient clinical signs of enteritis [6–9]. FCoV, thus, appears to be able to replicate primarily in the intestines with absence of viremia and without triggering a systemic immune response from the host and any significant effects on the RNA signature in whole blood. At organ level, however, a somewhat different picture was recently described by Malbon and colleagues [24]. In mesenteric lymph nodes (MLN) of FCoV-infected cats without clinical signs of FIP, many of the same processes observed in cats with FIP were also significantly upregulated at the RNA level when compared to FCoV-negative, clinically healthy cats. These included antiviral defense mechanisms, leukocyte chemotaxis, the IL-1 response, and interferon-related pathways. However, these changes were more strongly pronounced in cats with FIP than in those without. This led to the hypothesis that, in addition to the mere induction of these pathways, quantitative differences in gene expression might play a crucial role in disease development. Furthermore, the inflammatory triad of IL-1, IL-6, and TNF, along with CXCL10—a highly inflammatory chemokine—was found to be elevated exclusively in the MLN of cats with FIP. Taken together, these findings strongly support the view that antiviral treatment is not indicated in healthy cats that are infected with FCoV but do not develop FIP, as there is no clinical justification for such intervention. However, timely intervention and provision of GS-441524 to any cat developing the severe disease FIP is critical to save the animal.

We found that gene expression changes between cats with FIP at day 0 and both control groups (Kp and Kn cats) largely resembled the changes between day 0 and the first days of treatment, providing evidence for a normalization of the RNA signature early in treatment. Strikingly, cat 10, that had only been diagnosed with FIP incidentally during neutering, exhibited an RNA signature resembling healthy cats rather than cats with FIP before or during early treatment. This raises the question whether cat 10 was in an early disease stage and would have eventually developed full-blown FIP and thus benefited from early treatment or whether it would have been able to eliminate the infection on its own and would never have required treatment. Notably, we could not identify any gene marker for FIP that included cat 10 on day 0 but excluded healthy cats or other cats with FIP after the first week of treatment. For instance, while the monocyte/neutrophil-marker genes IFIT1, OAS1, LY6E, MX1, and OAS3 showed slightly increased RNA levels at day 0 for cat 10 and the B cell markers CD709A/B showed slightly reduced levels, they were neither elevated nor reduced, respectively, beyond RNA levels in some healthy cats or follow-up samples (Fig 3 a–c).

The RNA signature of day 0 cats was dominated by a general antiviral response, with strongly up-regulated genes enriched for ISGs, also up-regulated in other viral infections. It should be noted here that most functional annotation for cat genes is derived from orthology mapping from other species, mostly human and mouse, thus we may overlook feline-specific immune responses. Nevertheless, the core ISGs analyzed in this study were defined based on up-regulation in response to IFN across a diverse range of vertebrates (ranging from human to chicken), suggesting they indeed represent a vertebrate-wide response to viral infections, including in cats. Most up-regulated genes were already significantly reduced in expression by day 2 of treatment, indicating that switching off the FCoV-induced excessive immune response plays a crucial role in treatment success. Thus, although there might not be specific markers for FIP diagnosis, the present RNA-seq analysis suggests a wide range of potential biomarkers that could be used for monitoring treatment success. Some of these markers indicate a shift in cell type composition in cats with FIP from increased monocyte/neutrophil levels on day 0 to increased B cell levels from day 2. However, while the hematological results in part confirmed a change in cell type composition [31,38], the extent of changes in RNA levels was much more pronounced (Fig 4). This indicates a cumulative effect of gene expression changes within cells and changes in cell type composition. Since cell-type composition inferences were based on human-derived marker genes (i.e., from PanglaoDB), potential interspecies differences between cats and humans or mice introduce a degree of uncertainty in this observation. Moreover, many of the cell type marker genes identified as differentially expressed, in particular for monocytes and neutrophils, are not completely specific for these cell types according to the human protein atlas [50], but expressed among a wider range of immune cells. This further complicates the interpretation of shifts in cellular composition. Subsequent flow cytometric analysis of key markers will be required in follow-up studies to further explore these findings and evaluate their relevance on a phenotypic level. Moreover, an investigation of their respective response to other severe disease manifestations in cats, i.e., bacterial sepsis, will be necessary to evaluate their specificity. Nevertheless, the results of this study serve as a crucial milestone towards the identification of biomarkers for FIP to eventually support diagnosis and treatment monitoring.

A generalized lymphopenia is a common sign in cats with FIP, and despite strong individual variations, a severe decrease in all analyzed subsets, namely B cells, CD3 + T cells, NK cells and regulatory T cells (Tregs) is frequently observed [51–55]. It has been proposed that lymphocyte loss in FIP is caused by increased apoptosis, most likely due to the release of pro-apoptotic factors from virus-infected macrophages [52,53]. This is consistent with our observation that expression of B cell-specific markers (CD79A and CD79B) was strongly reduced on day 0 but during treatment returned to a normal range observed in healthy cats. During recovery due to antiviral treatment, most cats with lymphopenia before treatment not only reached normal lymphocyte counts but displayed a rebound effect by developing long-lasting mild to moderate lymphocytosis [31,38]. Notably, lymphocytosis has also been described in COVID-19 patients after treatment with Remdesivir, which resulted in a significant increase in B lymphocytes while T helper cells remained unaffected [56]. Flow cytometry-based lymphocyte subset analysis during treatment for some of the cats of this study revealed that lymphocytosis was caused by a significant increase of both CD4 + T-helper and CD21 + B cells [57,58]. Increased activation of B cells in cats with FIP has previously been described, leading to enhanced plasma-blast differentiation of B cells and increased plasma-blast numbers in whole blood [59]. If this activation of the B cell system involves B cell production in the bone marrow that persists during treatment, while FCoV-mediated lymphocyte apoptosis is halted by elimination of the virus, this could result in a transitional overproduction of B cells. The observed strong increase in CD4 + cells under antiviral treatment might also be a thymic counterreaction to virus-induced apoptosis. FIP has also been shown to be associated with a strong Treg suppression [60].

RNA signatures in cats with FIP rapidly normalized within the first week of treatment with only a few changes observed afterwards. These findings mirror the rapid clinical improvement of treated cats. In addition, they suggest that a substantially shorter duration of antiviral treatment might also be sufficient, as opposed to the previously established 84-days treatment protocol for the cats in this study. In a second treatment study we recently demonstrated equal effectiveness of a 42-day treatment duration compared to 84-days of treatment to achieve complete recovery from FIP [37]. However, the RNA-seq data shown here suggest that an even shorter treatment duration of only one month (28 days) might also be sufficient, as barely any further significant modifications in RNA signatures (only 36 DEGs) were observed when comparing day 28 to later timepoints (days 168, 252, and 336 after treatment start). Furthermore, clinical and laboratory parameters that are typically altered in cats with FIP [44,45] were within the corresponding reference range by day 28 at the latest, and no further relevant changes were observed beyond this timepoint [31]. Furthermore, FCoV, which was detectable in blood in 15 of the 18 cats before treatment (day 0), was no longer detectable in any cat from day 14 onwards [31]. Shorter treatment duration could offer considerable advantages. For cat owners, it would help alleviate both the financial and logistical burden associated with FIP treatment, given the high cost of GS-441524 and its limited legal availability in many countries worldwide, where access often relies on unregulated sources. For the animals, a shorter therapeutic course would reduce the cumulative stress of daily drug administration and limit exposure to potential adverse effects. One complication of GS-441524-associated urolithiasis, i.e., formation of stones in the urinary tract, was already reported for two cats undergoing treatment with GS-441524 and stone analysis confirmed that the stones were composed of 98% GS-441524 [61]. Therefore, a treatment approach reduced to the shortest effective duration will be of great practical advantage both from a clinical and animal humane point of view. It could enable the rescue of a much larger number of cats that currently cannot receive treatment—because of the above-mentioned reasons—and, therefore, still have to be euthanized. However, although the rapid normalization of RNA signatures is promising for a shorter treatment duration, further studies are needed to correlate these molecular changes with clinical endpoints in particular regarding relapse rates and long-term outcomes.

We conclude that RNA-seq analysis of whole blood samples during FIP treatment sheds new light on biological pathways involved in FIP before, during and after successful antiviral treatment. It highlighted key immune pathways involved in disease progression, treatment response, and recovery, in particular the role of an excessive and devastating immune response. DEGs identified in our analyses could serve as useful biomarkers for FIP diagnosis and treatment monitoring, pending further validation in additional trials. The rapid normalization of the blood RNA signature revealed by our study has exciting implications for more cost-effective shorter treatment of cats with FIP. Finally, the absence of significant changes in blood RNA signatures in cats with a non-pathogenic enteric FCoV infection is consistent with none or only very mild, transient clinical signs, further supporting that they do not require any therapeutic intervention.

## Materials and methods

### Prospective sample collection

In our previously published study, 18 cats with naturally occurring FIP were treated orally for 84 days with the multi-component drug Xraphconn^®^ provided by Mutian (Mutian Life Sciences Limited, China), containing the nucleoside analogue GS-441524 [31]. The diagnosis FIP in these cats was made according to the diagnostic tool of the Advisory Board on Cat Diseases (ABCD). The cats (referred to as “cats with FIP” for brevity in this study) had to have clinical and clinicopathological abnormalities typical for FIP [44,45]. In addition, FCoV infection had to be confirmed either by immunohistochemistry (IHC) from affected organs or by detection of FCoV by reverse transcriptase-quantitative PCR (RT-qPCR) from effusion, blood, or an organ fine needle aspirate. After the 84-day treatment period, follow-ups were performed at 3-month intervals for one year after treatment initiation [38]. Whole blood samples were collected at specific intervals: days 0, 2, 7, 28, 168 (24 weeks), 252 (36 weeks), and 336 (48 weeks) after the initiation of antiviral treatment. In addition, 5 healthy, single-housed cats, which were kept strictly indoors, were included as a “negative control group” (Kn cats), with blood samples collected at a single time point. These cats were tested anti-FCoV antibody-negative in serum and FCoV-RT-qPCR-negative in feces, based on previously established protocols [31,38,42].

The positive control group (Kp cats) comprised 12 cats that were cohabiting companion cats of the FIP study cats. At the time of sampling, all Kp cats were shedding FCoV in their feces and tested positive for anti-FCoV antibodies in serum [31,38,42]. Samples of healthy control cats (Kn cats and Kp cats) were collected at a single timepoint. At each timepoint (for the cats with FIP and Kn and Kp cats), 1 ml of whole blood was dropped directly from the vein via a cannula into a pre-prepared ‘homemade’ PAXgene RNA tube (4 ml cryovial tube). The homemade PAXgene tubes were prepared in a class 2 microbiological safety cabinet using sterile techniques with 2.76 ml of PAXgene RNA tubes reagent for intracellular RNA stabilization by PreAnalytiX (QIAGEN/ DB). Within 4 hours after collection, samples were stored at −80°C according to the manufacturer’s instructions until analysis.

For all cats (FIP, Kn, and Kp cats) and timepoints a complete blood cell count was performed using an automatic analyzer (Cell-Dyn 3500, Abbott Laboratories, IL, USA). A differential blood count was additionally performed manually on blood smears stained with Hema-Quick Stain/Diff-Quik stain (LT-SYS; Eberhard Lehmann) when hematology parameters were abnormal. Serum biochemistry parameters were measured using an automatic analyzer (Hitachi 911; Roche). The concentration of serum amyloid A (SAA) was determined using a latex agglutination turbidimetric immunoassay reaction (LZ Test SAA; Eiken Chemical) on a Cobas C501 clinical chemistry analyzer (Roche Diagnostics). For all cats, viral loads in both blood and fecal samples were quantified using a real-time TaqMan RT-qPCR assay, following previously published protocols and serum anti-FCoV antibody titers were assessed via indirect immunofluorescence assay (IFA), as also described before [31,38,42].

The study adhered to German guidelines for prospective studies and was approved by the Government of Upper Bavaria and the Ethical Committee of the Center for Clinical Veterinary Medicine at LMU Munich (reference numbers 55.2–2532.Vet_02-20-52 and 261-19-03-2021). All cat owners provided written informed consent prior to inclusion of their respective cats in the study. No methods involving sacrifice or anesthesia were required for any of the participating cats. Cats exhibiting pain solely attributable to FIP-related clinical signs at the initiation of the treatment study received adjunctive analgesic therapy as follows: buprenorphine (0.01 mg/kg; n = 4) or metamizole (30 mg/kg; n = 2) intravenously in cases of moderate pain; in one cat, due to more severe pain, pregabalin (2 mg/kg) was administered per os.

### RNA library preparation and sequencing

Total RNA was extracted with the QIAsymphony PAXgene Blood RNA Kit (Qiagen, Hilden, Germany) by the Core Facility Molecular Biology at the Center for Medical Research, Medical University of Graz, Austria. RNA quantity and quality were assessed using the NanoDrop 2000 Spectrophotometer (Thermo Fisher Scientific, Waltham, Massachusetts) and BioAnalyzer BA2100 (Agilent, Foster City, CA), respectively. All samples had an RNA Integrity Number (RIN) >7. 2 µg of isolated RNA underwent an additional DNAse I treatment using the RNA Clean & Concentrator-5 Kit (Zymo Research, Irvine, CA). Following the manufacturer’s instructions, RNA-Seq libraries were generated using the NEBNext^®^ Ultra™ II Directional RNA Library Prep Kit for Illumina® with the NEBNext^®^ rRNA depletion Module (both from New England Biolabs, Frankfurt am Main, Germany). After equimolar pooling of individual libraries, the pool was sequenced at the Vienna BioCenter Core Facilities (VBCF), Austria, on an Illumina NovaSeq 6000 platform with the 100 bp paired-end configuration. An average of 60 million reads per sample was obtained. RNA isolation of PAXgene tubes, library preparation and quality controls were performed.

### RNA-seq data analysis

Quality of the sequencing data was checked using FastQC (version 0.11.9) [62]. RNA-seq reads were mapped against the domestic cat genome assembly (version Felis catus 9.0), rRNA sequences and the FCoV genome (NC_002306.3) using ContextMap 2 (version 2.7.9) [63] (using BWA as short read aligner and with default parameters [64]). SAM output files of ContextMap2 were converted to BAM files using samtools [65]. Number of read fragments per gene were determined from BAM files in a strand-specific manner using featureCounts [66] and gene annotations from Ensembl (version 106). All these steps were implemented and run using the Watchdog workflow management system [67].

Differential gene expression analysis was performed using DESeq2 [68]. To determine gene expression changes during treatment, we used the following design to control for differences in individual cats: ~ cat + timepoint. P-values were adjusted for multiple testing using the method by Benjamini and Hochberg implemented in DESeq2 [69]. Functional enrichment analysis of GO terms and KEGG pathways was performed using the g:Profiler webserver [46] and the R package gprofiler2 [70], which provides an R interface to the webserver. We also evaluated enrichment for curated gene sets from MSigDB that are curated from various sources, including online pathway databases and the biomedical literature [71] and cell marker genes from PanglaoDB [49]. For the latter, cat genes were first mapped to human orthologs, since MSigDB and PanglaoDB provide only annotation for human and mouse genes. Vulcano plots, heatmaps and other figures were created in R. The Watchdog workflow and R scripts are available at https://doi.org/10.5281/zenodo.16611471.

## Supporting information

S1 Table Overview of collected full blood samples in cats with feline infectious peritonitis (FIP) during antiviral treatment course and positive control cats (Kp) and negative control cats (Kn). (DOCX)

S2 Table Overview of patients by showing the participating study cats with feline infectious peritonitis (FIP), including signalment, number of additional cats in the household, corresponding healthy partner cats (Kp cats), method of diagnosis of feline infectious peritonitis (FIP), FIP-associated signs, Xraphconn® treatment dose, other diseases at the start of and developing during treatment, adverse effects, and additional symptomatic therapy (table adapted from [31,42]). (DOCX)

S3 Table Log2 fold-changes for DEGs during treatment. (XLSX)

S4 Table Log2 fold-changes for DEGs for the comparison between day 0 and Kn and Kp cats. (XLSX)

S5 Table Functional enrichment for the 5 gene clusters from the treatment DEG analysis. (XLSX)
