# Supplementary material for: Whole blood RNA profiling in cats dissects the host immunological response during recovery from feline infectious peritonitis
Source: PLoS One. 2025 Sep 12;20(9):e0332248. doi: 10.1371/journal.pone.0332248 (PMC12431271; doi:10.1371/journal.pone.0332248)
Supplement: S1 Table — (DOCX) [file pone.0332248.s001.docx]

| Identification of treated FIP cats | Days after start of antiviral treatment | | | | | | | Kp^1^ (corresponding healthy, FCoV-shedding and FCoV-antibody-positive partner cat of the FIP cat) | Kn^2^ (healthy, not FCoV-shedding and FCoV-antibody-negative cats) |
| --- | --- | --- | --- | --- | --- | --- | --- | --- | --- |
|  | Day 0 | Day 2 | Day 7 | Day 28 | Day 168 | FU1 | FU2 |  |  |
| #001 | + |  | + | + | + |  |  | Kp010 |  |
| #002 | + | + | + | + | + | + | + | Kp012 |  |
| #003 | + |  | + | + | + |  |  |  |  |
| #004 | + | + | + | + | + | + | + | Kp011 |  |
| #005 |  |  | + | + | + | + | + | Kp001 |  |
| #006 | + | + | + | + | + | + | + |  |  |
| #007 | + |  | + | + | + | + | + |  |  |
| #008 | + | + | + | + | + | + | + |  |  |
| #009 | + | + | + | + | + |  |  |  |  |
| #010 | + | + | + | + | + | + |  |  |  |
| #011 | + | + | + | + | + | + | + | Kp006, Kp007, Kp008 |  |
| #012 | + | + | + | + | + | + | + | Kp009 |  |
| #013 | + | + | + | + | + | + | + |  |  |
| #014 | + | + | + | + | + | + | + | Kp002 |  |
| #015 | + | + | + | + | + | + | + | Kp003 |  |
| #016 | + | + | + | + | + | + | + | Kp004 |  |
| #017 | + | + | + | + | + | + | + |  |  |
| #018 | + | + | + | + | + | + | + | Kp005 |  |
| samples in total | 17 | 14 | 18 | 18 | 18 | 15 | 14 | 12 | 5 |

**S1 Table.** Overview of collected full blood samples in cats with feline infectious peritonitis (FIP) during antiviral treatment course, positive (Kp) and negative control cats (Kn).

FIP=feline infectious peritonitis; Kp=positive control cats; Kn=negative control cats

^1^positive control samples consisted of 12 cats that were healthy cohabitating partner cats (without FIP) of the treatment study cats with FIP. At the time of sampling, all 12 cats were shedding FCoV in their feces and had detectable anti-FCoV antibody titers in serum

^2^negative control samples were obtained at a single time point from 5 healthy, single-kept indoor-only cats; these cats were tested negative for anti-FCoV antibodies in serum and showed no fecal shedding of FCoV
