# Supplementary material for: Whole blood RNA profiling in cats dissects the host immunological response during recovery from feline infectious peritonitis
Source: PLoS One. 2025 Sep 12;20(9):e0332248. doi: 10.1371/journal.pone.0332248 (PMC12431271; doi:10.1371/journal.pone.0332248)
Supplement: S2 Table — (DOCX) [file pone.0332248.s002.docx]

**S2 Table.** Table listing participating study cats with feline infectious peritonitis (FIP), including signalment, number of additional cats in the household, corresponding healthy partner cats (Kp cats), method of diagnosis of feline infectious peritonitis (FIP), FIP-associated signs, Xraphconn^®^ treatment dose, other diseases at the start of and developing during treatment, adverse effects, and additional symptomatic therapy (table adapted from (31,42)).

| **Cat with FIP** | **Age (months) at time of study inclusion** | **Additional cats in the household (number)** | **Positive control partner cat (Kp)** | **Associated study day (after treatment initiation) of the partner cat with FIP, on which the samples of each Kp were collected** | **FIP-associated cardinal signs** | **Dosage** | **Adverse effects and duration (on days of treatment)** | **Additional symptomatic therapy** |
| --- | --- | --- | --- | --- | --- | --- | --- | --- |
|  |  |  |  |  |  | **(mg/kg q 24h)** |  |  |
| #001 | 6.0 | yes (1) | Kp010 | 145 | ocular signs | 10 mg/kg | lymphocytosis (2–end^1^) | fluid therapy^2^, metamizole^3^ |
|  |  |  |  |  |  |  |  |  |
| #002 | 6.3 | yes (1) | Kp012 | 143 | neurologic signs, ocular signs | 10 mg/kg | lymphocytosis (2–end) | fluid therapy, antibiotics^4^, buprenorphine^5^ |
| #003 | 9.8 | yes (1) |  |  | abdominal effusion | 5 mg/kg | increased liver enzyme activity (7–14) | fluid therapy, antibiotics, maropitant^6^, mirtazapine^7^ |
| #004 | 7.2 | yes (1) | Kp011 | 135 | abdominal effusion | 5 mg/kg | lymphocytosis (2–end), | fluid therapy, metamizole-sodium, antibiotics, maropitant, mirtazapine |
|  |  |  |  |  |  |  | eosinophilia (14–56) |  |
| #005 | 6.4 | yes (1) | Kp001 | 83 | abdominal effusion | 5 mg/kg | lymphocytosis (7–end), | fluid therapy, antibiotics |
|  |  |  |  |  |  |  | increased liver enzyme activity (4–28), |  |
|  |  |  |  |  |  |  | eosinophilia (14–end) |  |
| #006 | 10.7 | yes (1) |  |  | abdominal effusion | 5 mg/kg | lymphocytosis (83–end) | antibiotics, mirtazapine |
| #007 | 4.7 | yes (1) |  |  | abdominal effusion, thoracic effusion | 5 mg/kg | lymphocytosis (4–end), | fluid therapy, antibiotics, silymarin^8^ |
|  |  |  |  |  |  |  | increased liver enzyme activity (4–83), |  |
|  |  |  |  |  |  |  | eosinophilia (14–end) |  |
| #008 | 6.4 | no |  |  | thoracic effusion | 5 mg/kg | lymphocytosis (7), | fluid therapy, antibiotics, oxygen cage^9^ |
|  |  |  |  |  |  |  | eosinophilia (14–83) |  |
| #009 | 8.9 | yes (3) |  |  | abdominal effusion | 5 mg/kg | lymphocytosis (28–end), | fluid therapy, antibiotics, mirtazapine, silymarin |
|  |  |  |  |  |  |  | increased liver enzyme activity (28–83), |  |
|  |  |  |  |  |  |  | eosinophilia (28–end) |  |
| #010 | 39.1 | yes (3) |  |  | abdominal effusion | 5 mg/kg | eosinophilia (2–end) |  |
| #011 | 56.5 | yes (3) | Kp006, Kp007, Kp008 | 83 | abdominal effusion | 5 mg/kg | lymphocytosis (4–14), | fluid therapy, antibiotics |
|  |  |  |  |  |  |  | increased liver enzyme activity (4–97) |  |
| #012 | 11.7 | yes (1) | Kp009 | 83 | thoracic effusion | 5 mg/kg | lymphocytosis (2–end), | fluid therapy, antibiotics, buprenorphine |
|  |  |  |  |  |  |  | increased liver enzyme activity (28–56), |  |
|  |  |  |  |  |  |  | eosinophilia (28–83) |  |
| #013 | 28.8 | yes (9) |  |  | thoracic effusion | 5 mg/kg | eosinophilia (28–end) | fluid therapy, antibiotics |
| #014 | 7.5 | yes (1) | Kp002 | 56 | abdominal effusion | 5 mg/kg |  | fluid therapy, antibiotics maropitant, mirtazapine, buprenorphine, pregabalin^10^ |
| #015 | 7.6 | yes (1) | Kp003 | 56 | abdominal effusion | 5 mg/kg | lymphocytosis (2–56), | fluid therapy, antibiotics, buprenorphine, meloxicam^11^ |
|  |  |  |  |  |  |  | eosinophilia (2–28) |  |
| #016 | 8.9 | yes (1) | Kp004 | 56 | abdominal effusion | 5 mg/kg | lymphocytosis (7–14), | fluid therapy, mirtazapine |
|  |  |  |  |  |  |  | increased liver enzyme activity (2–14) |  |
| #017 | 77.7 | yes (1) |  |  | thoracic effusion | 5 mg/kg | lymphocytosis (2–end), | fluid therapy, antibiotics |
|  |  |  |  |  |  |  | eosinophilia (28–end) |  |
| #018 | 7.6 | yes (2) | Kp005 | 56 | abdominal effusion | 5 mg/kg | lymphocytosis (4–end), | fluid therapy, antibiotics |
|  |  |  |  |  |  |  | eosinophilia (2–end) |  |
